# Proton Pump Inhibitors and Cancer: Current State of Play

**DOI:** 10.3389/fphar.2022.798272

**Published:** 2022-03-14

**Authors:** Marie Bridoux, Nicolas Simon, Anthony Turpin

**Affiliations:** ^1^ University of Lille, Lille, France; ^2^ Medical Oncology Department, Lille University Hospital, Lille, France; ^3^ CHU Lille, ULR 7365—GRITA—Groupe de Recherche sur les Formes Injectables et les Technologies Associées, University of Lille, Lille, France; ^4^ Medical Oncology Department, CNRS, Inserm, CHU Lille, Institut Pasteur de Lille, UMR9020—UMR-S 1277—CANTHER-Cancer Heterogeneity, Plasticity and Resistance to Therapies, CHU Lille, University of Lille, Lille, France

**Keywords:** cancer, proton-pump inhibitors, chemotherapy, targeted therapies, drug interactions

## Abstract

**Background:** Proton pump inhibitors (PPIs) are one of the most widely used drugs worldwide and are overprescribed in patients with cancer; there is increasing evidence of their effects on cancer development and survival. The objective of this narrative review is to comprehensively identify cancer medications that have clinically meaningful drug–drug interactions (DDIs) with PPIs, including loss of efficacy or adverse effects, and to explore the association between PPIs and cancer.

**Methods:** A PubMed search of English language studies published from 1 January 2016, to 1 June 2021 was conducted. The search terms included “proton pump inhibitors,” “cancer,” “chemotherapy,” “immunotherapy,” “hormonotherapies,” “targeted therapies,” “tyrosine kinase inhibitors,” and “gut microbiome”. Recent and relevant clinical trials, meta-analyses, and reviews were included.

**Results:** PPIs may have pro-tumor activity by increasing plasma gastrin levels or anti-tumor activity by inhibiting V-ATPases. However, their impact on cancer survival remains unclear. PPIs may decrease the efficacy of some antineoplastic agents through direct DDIs (e.g., some tyrosine kinase inhibitors, capecitabine, irinotecan, methotrexate). More complex DDIs seem to exist for immunotherapies with indirect interactions through the microbiome. PPIs worsen hypomagnesemia, bone loss, iron, and vitamin B12 deficiencies but may have a protective effect on the renal system.

**Discussion/Conclusions:** PPIs may interact with the cancer microbiome and the efficacy of various antineoplastic agents, although only a few DDIs involving PPIs are clinically significant. Further pharmaco-epidemiological studies are warranted, but physicians should be aware of the potential consequences of PPI use, which should be dose appropriate and prescribed according to guidelines.

## Introduction

Proton pump inhibitors (PPIs) are one of the most commonly prescribed drugs worldwide (Kinoshita et al., 2018). These drugs irreversibly inhibit H+/K+ adenosine triphosphatase pumps in gastric parietal cells and results in the suppression of gastric acid production for >24 h (Yibirin et al., 2021).

The US Food and Drug Administration (FDA) has approved PPIs for a variety of gastric acid‐related conditions, including gastroesophageal reflux disease (GERD), duodenal or gastric ulcers, Helicobacter pylori infections, and Zollinger-Ellison syndrome as well as the prevention of nonsteroidal anti-inflammatory drug (NSAID)-associated gastrointestinal lesions in at-risk patients (aged > 65 years, with a history of gastrointestinal ulcer or with concomitant antiplatelet, anticoagulant, or corticosteroid therapy) (Strand et al., 2017). Long-term treatment is usually required for many of these disorders, which increases the potential for clinically significant drug interactions in patients. In addition, off-label prescribing has been widely reported, particularly in functional dyspepsia and in the prevention of NSAID-induced gastroduodenal lesions in non-at-risk patients (Lassalle et al., 2020).

The use of PPIs has grown in many countries since their market introduction in the late 1980s. For instance, in France, more than 15 million people with health insurance, or almost one-third of the French adult population, were PPI users in 2015 (Singh et al., 2018; Lassalle et al., 2020). In one study, PPI indication could not be established for one-third of the patients, and no measurable risk factor was found for three-quarters of the prophylactic prescriptions associated with NSAIDs (Lassalle et al., 2020). Approximately 20% of patients with cancer use PPIs (Kinoshita et al., 2018; Tvingsholm et al., 2018; Sharma et al., 2019); however, PPIs are often overprescribed in these patients to treat side effects of chemotherapy such as GERD or as prophylaxes in combination therapy with corticosteroids or NSAIDs (Lassalle et al., 2020).

In general, PPIs are believed to have few adverse events, as they are generally well tolerated. However, PPIs have been reported to be associated with gastrointestinal disorders (nausea, abdominal pain, transit disorder), ionic absorption disorders (hypomagnesemia, iron deficiency, vitamin B12 deficiency), kidney failure, infections (pneumonia, Clostridium difficile infections, peritonitis), and bone fractures (Singh et al., 2018; Yibirin et al., 2021).

In addition, PPIs are involved in various drug–drug interactions (DDIs) (Wedemeyer and Blume, 2014; Strand et al., 2017; Patel et al., 2020; Uchiyama et al., 2021). By elevating gastric pH, PPIs influence the absorption of gastric pH-dependent drugs. Indeed, an increase in the gastric pH of some weakly basic drugs results in decreases in dissolution and subsequent absorption rates (Wedemeyer and Blume, 2014; Patel et al., 2020). PPIs could potentially also affect drug elimination, as they are potential inhibitors of organic cation transporters (OCTs, which are involved in renal excretion of substrate medications) and P-glycoprotein efflux transporters (Wedemeyer and Blume, 2014; Patel et al., 2020). PPIs are predominantly metabolized in the liver by the cytochrome P450 enzyme (CYP) system, mainly by CYP2C19 and CYP3A4 (Wedemeyer and Blume, 2014). They have the ability to act either as inhibitors or inducers of CYP; the inhibition of CYP increases systemic exposure to a drug (Patel et al., 2020). Omeprazole has considerable DDI potential because of its high affinity for CYP2C19 and moderate affinity for CYP3A4 (Wedemeyer and Blume, 2014). Esomeprazole also inhibits CYP2C19 to a clinically significant degree, whereas CYP2C19 inhibition by other PPIs is not clinically relevant (Patel et al., 2020).

However, only a few DDIs involving PPIs are clinically significant (Wedemeyer and Blume, 2014). Nonetheless, the risk of drug interactions should be considered when choosing a PPI to treat gastric acid-related disorders.

**FIGURE 1:**
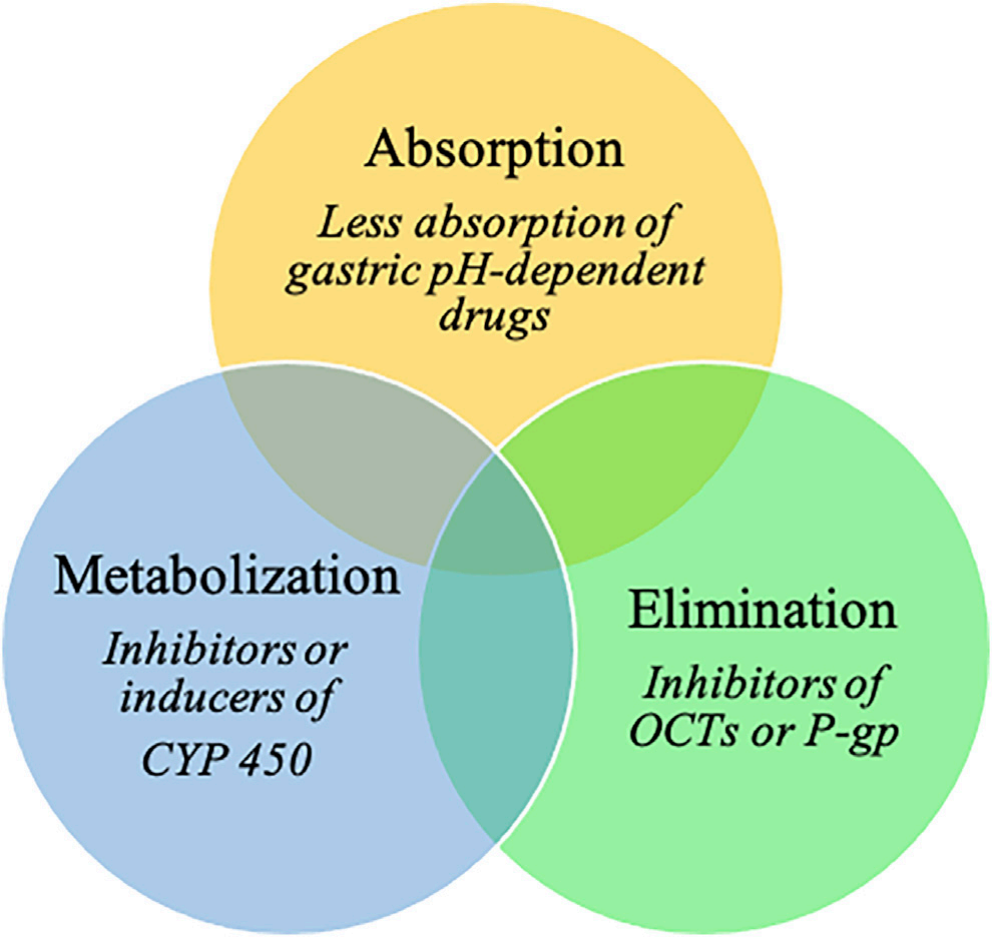
Pharmacodynamic mechanisms of PPIs drug-drug interactions.

PPIs may be involved in many interactions with cancer and cancer-related treatments (Figure 1). Thus, our aims are (i) to comprehensively address the impact of PPI use on cancer occurrence and outcomes and (ii) to pragmatically identify cancer drugs that have clinically meaningful DDIs with PPIs, including loss of efficacy or adverse events.

## Review

### Methods

This narrative review, with expert opinion, is based on the literature published from 1 January 2016, to 1 June 2021. PubMed searches were limited to English language studies. The search used the following keywords: “proton pump inhibitors,” “cancer,” “chemotherapy,” “immunotherapy,” “hormonotherapies,” “targeted therapies,” “tyrosine kinase inhibitors,” and “gut microbiome” (detailed list in Supplementary Table S1). The search was extended beyond 5 years for specific terms without relevant data in the last 5 years (detailed list in Supplementary Table S2). We selected recent and relevant studies, including clinical trials, meta-analyses, and reviews. Letters to the editor and congress communications were excluded. A total of 98 articles were included in this review.

### Results

#### Cancer Occurrence and Outcomes

One of the hallmarks of cancer is deregulation of the energetic metabolism of tumor cells (Hanahan and Weinberg, 2011). Tumor cells activate the aerobic glycolysis pathways to perform their biosynthesis, which generates an excess of protons and lactates in the intracellular space. V-ATPases are vacuolar proton pumps that maintain a neutral intracellular sector by increasing the acidity of the extracellular medium. These pumps are overexpressed in tumor cells and increase the acidity of the tumor microenvironment, which is believed to be involved in tumorigenesis, tumor proliferation, tumor progression, tumor invasion, and treatment resistance (Whitton et al., 2018; Tozzi et al., 2020).

Several studies have shown that PPIs inhibit V-ATPases *in vitro* and *in vivo* (Ikemura et al., 2017a; Tozzi et al., 2020). By decreasing the acidity of the tumor microenvironment, inhibition of V-ATPases slows cell proliferation and induces tumor cell apoptosis. Therefore, PPIs may have anti-tumor activity of their own and may increase the efficacy of anti-tumor therapies via V-ATPase inhibition (Ikemura et al., 2017a; Tvingsholm et al., 2018; Tozzi et al., 2020).

In contrast, PPI administration increases plasma gastrin levels (Kinoshita et al., 2018). Since gastrin promotes the proliferation of gastric enterochromaffin-like cells, PPIs can stimulate the development of gastric neuroendocrine and carcinoid tumors (Kinoshita et al., 2018). Similarly, some observational studies have suggested that PPIs may increase the risk of digestive cancers, such as esophageal, gastric, pancreatic, and colorectal cancer (Brusselaers et al., 2017; Hwang et al., 2017; Kearns et al., 2017; Brusselaers et al., 2018; Kinoshita et al., 2018).

Moreover, PPIs may also affect the prognosis of patients with cancer, but there are contradictory results regarding this issue (Papagerakis et al., 2014; Graham et al., 2016; Kearns et al., 2017; Tvingsholm et al., 2018; Wu et al., 2019). Table 1 summarizes the major studies on cancer-specific mortality among PPI users.

**TABLE 1:** Main studies on cancer-specific mortality among proton pump inhibitor users.

Location	Type of study	PPI intake definition	Number of patients	Mortality risk	References
All (except non‐melanoma skin cancer)	Retrospective—registry	≥2 prescriptions within 6 months following the cancer diagnosis	Users = 36,066 vs. non-users = 311,853	Higher: HR = 1.29, 95%CI 1.27–1.32	Tvingsholm et al. (2018)
All	Retrospective - U.S. electronic health data	≥1 prescription of omeprazole in the electronic health record data	Not specified	Lower: HR = 0.9, 95%CI 0.84–0.96	Wu et al. (2019)
Colorectal cancer	Retrospective	Use of PPIs at the time of the oncology consultation	Users = 117 *vs*. non-users = 1,187	Higher: HR = 1.343, 95%CI 1.011–1.785, *p* **=** 0.042	Graham et al., (2016)
Pancreatic cancer	Retrospective	Short-term active PPI users (first prescription <12 months and most recent prescription <6 months prior to index rate)	Users = 1,109 *vs.* non-users = 3,004	Higher: HR = 1.11, 95%CI 1.02–1.21	Kearns et al. (2017)
Head and neck cancers	Retrospective	≥1 PPI usage documented after diagnosis date	Users = 327 *vs.* non-users = 269	Lower: HR = 0.55, 95%CI 0.40–0.74, *p* < 0.0001	Papagerakis et al. (2014)

HR: Hazard ratio; PPI: proton pump inhibitors.

#### Modulation of Cancer-Related Treatments Side Effects

PPI co-medication may enhance the side effects induced by some anti-tumor treatments.

Clinically, PPIs increase bone loss, which is a risk factor for fractures (Mazziotti et al., 2010; Panday et al., 2014). Therefore, hormonotherapies such as aromatase inhibitors, used in breast cancer, and androgen deprivation therapy, used in prostate cancer, cause bone loss and increase the risk of fractures (Mazziotti et al., 2010; Panday et al., 2014). With concomitant use, the risk of fractures may increase.

Biologically, PPIs might worsen hypomagnesemia induced by therapies such as cisplatin, anti-epithelial growth factor receptor (EGFR) monoclonal antibodies, or tyrosine kinase inhibitors (TKIs) (Abu-Amna and Bar-Sela, 2019). In addition, reduction of gastric acidity decreases the absorption of ferrous iron and vitamin B12, which may lead to anemia (Singh et al., 2018).

In contrast, PPIs may have a renal protective effect by inhibiting OCT2, a renal proximal tubular transmembrane transporter involved in renal elimination of cisplatin (Ikemura et al., 2017a). Its inhibition by PPIs decreases renal accumulation and cisplatin-induced nephrotoxicity. This protective effect was found in a retrospective study involving patients treated with cisplatin and 5-fluorouracil for cancer of the upper aerodigestive tract (Ikemura et al., 2017b). A phase III trial investigating the protective effect of pantoprazole on cisplatin-induced nephrotoxicity in upper aerodigestive tract cancers is currently underway (NCT04217512).

#### Modulation of Cancer-Related Treatments Efficacy

##### Oral Chemotherapeutic Agents

Capecitabine is an oral prodrug of 5-fluorouracil, commonly used in digestive and breast cancers, with optimal absorption under acidic conditions (capecitabine dissociation constant pKa = 1.92). It has been speculated that an increase in the gastric pH may lead to reduced dissolution and absorption of capecitabine tablets, although *in vitro* data have not confirmed this to date (Cheng et al., 2019; Sekido et al., 2019). However, several studies on colorectal cancer have shown poorer survival when PPIs are combined with capecitabine compared with capecitabine monotherapy (Sun et al., 2016; Chu et al., 2017; Rhinehart et al., 2018; Wong et al., 2019; Kim et al., 2021). Table 2 summarizes the results of the main studies on this topic.

**TABLE 2:** Main studies on interaction of capecitabine and co-medication with proton pump inhibitors.

Location and Stage	Type of study	Treatment	PPI intake definition	Number of patients	Results	Reference
All stages colorectal cancer	Retrospective	Capecitabine monotherapy	PPI documented on medication list ≥20% of the treatment duration	N = 70	Reduced PFS: HR = 2.24, 95%CI 1.06–4.41	Rhinehart et al. (2018)
Early colorectal cancer (stage I to III)	Retrospective	Capecitabine monotherapy	PPIs documented on prescription refill data at any point in time during treatment	N = 298	Reduced 5-year RFS rate: HR = 1.83, 95%CI 1.07–3.35, *p* = 0.03	Sun et al. (2016)
Early colorectal cancer (stage II to III)	Retrospective	CAPOX versus FOLFOX	PPIs documented on prescription refill data at any time during treatment	N = 389	Reduced 3-year RFS in CAPOX-treated patients: HR 2.03, 95%CI 1.06–3.38 but not among FOLFOX-treated patients: HR 0.51, 95%CI 0.25–1.06	Wong et al. (2019)
Metastatic gastroesophageal cancer	Secondary analysis of multicentric randomized TRIO-013/LOGiC trial	CAPOX	≥20% overlap between PPI prescription and treatment duration	N = 545	Reduced PFS: HR 1.55, 95%CI 1.29–1.81, *p* < 0.001 and reduced OS: HR = 1.34, 95%CI 1.06–1.62, p = 0.04	Chu et al. (2017)
Metastatic colorectal cancer	Post hoc analysis from the AXEPT phase III randomized trial	mXELIRI versus FOLFIRI	≥20% overlap between use of any PPI and treatment duration	N = 482	Not significantly reduced OS: HR = 1.83, 95%CI 0.96–3.48 and PFS: HR = 1.73, 95%CI 0.94–3.21	Kim et al. (2021)

CAPOX, capecitabine and oxaliplatin, FOLFIRI, leucovorin, fluorouracil, and irinotecan; FOLFOX, leucovorin, fluorouracil, and oxaliplatin; HR, hazard ratio; OS, overall survival; PPI, proton pump inhibitors; PFS, progression-free survival; RFS, recurrence-free survival; modified XELIRI, capecitabine, and irinotecan.

Cyclophosphamide is metabolized by CYP2C19 (Griskevicius et al., 2003). Since PPIs are competitive inhibitors of CYP2C19, DDIs may decrease its efficacy. However, no clinical trials have explored their DDIs.

Other commonly used oral chemotherapeutic agents include etoposide, temozolomide, topotecan, and vinorelbine. There are no DDIs between these drugs and PPIs described in the present literature.

##### Intravenous Chemotherapeutic Agents

PPIs are also thought to be involved in DDIs with two intravenous agents, irinotecan, a topoisomerase I inhibitor, and methotrexate, an antifolate agent. One of the mechanisms of resistance to irinotecan is the rapid degradation of topoisomerase I. Topoisomerase I degradation occurs in the proteasome following phosphorylation by DNA-PKc. CTDSP1 nuclear phosphatase is believed to negatively regulate the activation of DNA-PKc. Therefore, high expression of CTDSP1 inhibits DNA-PKc activation and limits topoisomerase I degradation (Matsuoka et al., 2020). PPIs such as rabeprazole inhibit the activity of CTDSP1. Consequently, DNA-PKc is activated, and the degradation of topoisomerase I is enhanced. A retrospective study found a poor clinical response to irinotecan in patients with colorectal cancer when used in combination with rabeprazole (Matsuoka et al., 2020). However, in a pharmacological study, omeprazole co-medication did not affect the main pharmacokinetic parameters of irinotecan and its main metabolites (van der Bol et al., 2011). The observed changes may be related to mechanisms other than pharmacokinetic alterations.

High-dose methotrexate, usually defined as >1 g/m^2^, is widely used to treat a variety of malignancies, including lymphoma, acute leukemia, and osteosarcoma (Bezabeh et al., 2012). Methotrexate is eliminated by active tubular secretion through the organic anion transporter 3 (OAT3) (Narumi et al., 2017). PPIs may inhibit OAT3 and therefore decrease methotrexate clearance, resulting in elevated serum levels of methotrexate and its metabolite hydroxymethotrexate and may induce methotrexate toxicity (Bezabeh et al., 2012). However, the mechanism of interaction is not well understood, and current data remain controversial regarding this DDI (Ranchon et al., 2018; Wang et al., 2020). The FDA recommends that clinicians “use caution when administering high-dose methotrexate to patients receiving proton pump inhibitor therapy” (Bezabeh et al., 2012).

No DDIs were found between PPIs and other parenteral chemotherapies.

##### Targeted Therapies

A well-known DDI between PPIs and cancer treatment concerns several TKIs. TKIs are oral antineoplastic treatments used in various solid and hematological tumors. By increasing the gastric pH, PPIs decrease the absorption of some TKIs. TKIs are weak bases and can be present in either the ionized or non-ionized form according to the pH in the stomach. When a TKI is co-administered with a PPI, the pH in the stomach rises from 1 to 4, and the equilibrium of ionized and non-ionized drugs shifts to the less soluble non-ionized form, resulting in a decrease in the bioavailability of the TKI (van Leeuwen et al., 2014).

Several studies have investigated the DDIs between PPIs and TKIs; the results are contradictory concerning PPI interaction with the bioavailability and activity of TKIs. A summary of these DDIs is presented in Table 3 (Rugo et al., 2005; Egorin et al., 2009; Yin et al., 2010; Yin et al., 2012; Takahashi et al., 2012; Hilton et al., 2013; Abbas et al., 2013; Musib et al., 2013; Tan et al., 2013; Johansson et al., 2014; Narasimhan et al., 2014; Chu et al., 2015; Kletzl et al., 2015; Ha et al., 2015; Iurlo et al., 2016; Kumarakulasinghe et al., 2016; Lam et al., 2016; Zenke et al., 2016; Chu et al., 2017; Lalani et al., 2017; Lacy et al., 2017; Lau et al., 2017; Morcos et al., 2017; Yokota et al., 2017; de Jong et al., 2018; Nieves Sedano et al., 2018; Ohgami et al., 2018; Fang et al., 2019; Mir et al., 2019; Vishwanathan et al., 2018; Pisano et al., 2019; de Man et al., 2019; Koutake et al., 2020; Ruanglertboon et al., 2020; Van De Sijpe et al., 2020; Chen et al., 2021; Rassy et al., 2021). Table 4 summarizes the main studies reporting reductions in the survival of patients receiving this combination of medication (Chu et al., 2015; Ha et al., 2015; Fang et al., 2019; Mir et al., 2019).

**TABLE 3:** Summary of drug–drug interactions between proton pump inhibitors and tyrosine kinase inhibitors.

Molecule	Target	Type of cancer	Impact on bioavailability	Impact on survival	Label recommendation
Afatinib	EGFR	NSCLC	−	−	−
Alectinib	ALK	NSCLC	✓ no effect Morcos et al., (2017)	−	−
Axitinib	VEGFR	RCC	✓ no effect Rugo et al., (2005)	✓ no effect Lalani et al. (2017)	−
Bosutinib	Bcr-Abl	CML	✗ reduced Abbas et al. (2013)	−	✗ caution (consider antacids)
Brigatinib	ALK	NSCLC	−	−	−
Cabozantinib	VEGFR	HCC, RCC	✓ no effect Lacy et al., (2017)	✓ no effect Rassy et al. (2021)	−
Ceritinib	ALK	NSCLC	✓ no effect Lau et al., (2017)	−	−
Cobimetinib	MEK	Melanoma	✓ no effect Musib et al. (2013)	−	✓
Crizotinib	ALK, ROS1	ALCL, NSCLC	−	−	✓
Dabrafenib	BRAF	Melanoma, NSCLC	−	−	−
Dasatinib	Bcr-Abl	ALL, CML	✗ reduced Takahashi et al. (2012)	✓ no effect Koutake et al., (2020)	✗ avoid (consider antacids)
Encorafenib	RAF	CRC, melanoma	−	−	✓
Erlotinib	EGFR	NSCLC	? no effect Hilton et al. (2013) or reduced Kletzl et al. (2015); Ohgami et al. (2018)	? no effect Kumarakulasinghe et al. (2016); Zenke et al. (2016) or reduced PFS Chu et al. (2015); Lam et al. (2016); Nieves Sedano et al. (2018) and OS Chu et al. (2015)	✗ avoid
Gefitinib	EGFR	NSCLC	✗ reduced Yokota et al. (2017)	? no effect Kumarakulasinghe et al. (2016); Zenke et al. (2016) or reduced PFS Nieves Sedano et al. (2018) and OS Fang et al. (2019)	✗ avoid
Ibrutinib	BTK	CLL, lymphoma			−
Imatinib	Bcr-Abl, Kit	ALL, CML, DFSP, GIST, HES, MDS	✓ no effect Egorin et al. (2009)	✓ no effect Iurlo et al. (2016)	✓ no effect de Jong et al. (2018)
Lapatinib	HER2	Breast cancer	−	Unclear Chu et al. (2017)	−
Lenvatinib	FGFR, Kit, VEGFR	HCC, thyroid cancer, RCC	−	−	−
Lorlatinib	ALK, ROS1	NSCLC	✗ reduced Chen et al., (2021)	−	−
Nilotinib	Bcr-Abl, Kit	CML, GIST	✓ no significant Yin et al., (2012) or modest effect Yin et al. (2010)	✓ no effect Yin et al. (2012)	✗ caution
Osimertinib	EGFR	NSCLC	✓ no effect Vishwanathan et al. (2018)	−	−
Pazopanib	Kit, VEGFR	RCC, STS	✗ reduced Tan et al. (2013)	? no effect Van De Sijpe et al., (2020) or reduced PFS Mir et al., 2019 Pisano et al., (2019) and OS Mir et al. (2019)	−
Ponatinib	Bcr-Abl, Kit, VEGFR	ALL, CML	✓ no significant effect Narasimhan et al. (2014)	−	✓
Regorafenib	EGFR, Kit, VEGFR	CRC, GIST, HCC	✓ no effect de Man et al. (2019)	−	−
Ruxolitinib	JAK	myelofibrosis	−	−	−
Sorafenib	Kit, RAF, VEGFR	HCC, RCC, thyroid cancer	−	✓ no effect Lalani et al. (2017); Ruanglertboon et al. (2020)	✓
Sunitinib	Kit, VEGFR	GIST, PNET, RCC	−	? no effect Lalani et al. (2017) or reduced PFS and OS Ha et al. (2015)	−
Trametinib	MEK	Melanoma, NSCLC	−	−	−
Vandetanib	EGFR, VEGFR	Thyroid cancer	✓ no effect Johansson et al. (2014)	−	−
Vemurafenib	BRAF	Melanoma	−	−	−

ALCL, anaplastic large cell lymphoma; ALL, acute lymphoblastic leukemia; ALK, anaplastic lymphoma kinase; BTK, Bruton’s tyrosine kinase; CML, chronic myeloid leukemia; CLL, chronic lymphocytic leukemia; CRC, colorectal cancer; DFSP, dermatofibrosarcoma protuberans; EGFR, epidermal growth factor receptor; GIST, gastrointestinal stromal tumor; HCC, hepatocellular carcinoma; HER2, human epidermal growth factor receptor 2; HES, hypereosinophilic syndrome; JAK, Janus kinase; MDS, myelodysplastic syndrome; NSCLC, non-small cell lung cancer; OS, overall survival; PFS, progression-free survival; PNET, pancreatic neuroendocrine tumors; RCC, renal cell carcinoma; STS, soft tissue sarcoma; VEGFR, vascular endothelial growth factor receptor. ✓ coadministration shows no interaction, ✗ coadministration is not recommended, ? differing effects, - no information available.

**TABLE 4:** Main studies reporting a decrease in survival of patients receiving proton pump inhibitors with tyrosine kinase inhibitors.

Target	Molecule	Type of study	PPI intake definition	Number of patients	Results	Ref
EGFR	Gefitinib	Retrospective–nationwide cohort	≥1 prescription of PPIs. High coverage ratio if >20% overlap between PPIs and gefitinib	*N* = 1,278	Reduced OS (lower PPI coverage ratio HR = 1.29, 95%CI 1.03–1.62, *p* = 0.027; higher PPI coverage ratio HR = 1.67, 95%CI 1.33–2.09, *p* < 0.001)	Fang et al. (2019)
EGFR	Erlotinib	Retrospective	≥20% overlap between PPIs and erlotinib	*N* = 507	Reduced PFS (HR = 1.83, 95%CI 1.48–2.25) and OS (HR = 1.37, 95%CI 1.11–1.69)	Chu et al. (2015)
VEGF	Sunitinib	Retrospective	PPIs continuously throughout sunitinib therapy	*N* = 231	Reduced PFS (*p* = 0.04) and OS (*p* = 0.02)	Ha et al. (2015)
VEGF	Pazopanib	Supplementary analysis of single-arm phase II and placebo-controlled phase III studies	PPIs during treatment duration	*N* = 333	Reduced PFS (HR = 1.49, 95%CI 1.11–1.99, *p* = 0.01) and OS (HR = 1.81, 95%CI 1.31–2.49, *p* < 0.01)	Mir et al. (2019)

EGFR, epidermal growth factor receptor; HR, hazard ratio; OS, overall survival; PPI, proton pump inhibitors; PFS, progression-free survival; RFS, recurrence-free survival; VEGF, vascular endothelial growth factor.

In particular, another DDI concerns PPIs and cyclin-dependent kinases (CDKs), which are major enzymes that control the cell cycle and cell division. CDK 4/6 inhibitors, such as palbociclib and ribociclib, have been used with success to treat breast cancer. Palbociclib is a weak base with gastric pH-dependent solubility, and PPIs decrease their bioavailability under fasting conditions. However, the impact of PPIs on the bioavailability of palbociclib is mitigated by food intake (Sun et al., 2017). No study has investigated the interaction between PPIs and palbociclib on survival. However, PPIs do not affect the bioavailability of ribociclib (Samant et al., 2018).

There are no data or clinical relevance regarding any interaction between PPIs and targeted therapies such as mTOR inhibitors, PARP inhibitors, or PI3K inhibitors (Patel et al., 2020; Uchiyama et al., 2021).

##### Hormonotherapy

No interaction between PPIs and second-generation antiandrogens, such as abiraterone acetate or enzalutamide, has been described. However, DDIs may occur in patients with prostate cancer because of the inhibition of CYP2C8 and 2D6 by abiraterone and induction of CYP3A4, 2C9, and 2C19 by enzalutamide (Del Re et al., 2017). As CYP2C19, and to a lesser degree CYP3A4, clear the PPIs metabolically (Ward and Kearns, 2013), there is a potential for DDIs between PPIs and enzalutamide or apalutamide. Further studies are needed to address this topic.

No DDI is described between PPIs and breast cancer endocrine therapies.

##### Immunotherapy

There is growing evidence that the gut microbiome has a central role in controlling both the antitumor immune response in digestive organs and the host immune system response to anti-cancer therapies (Gopalakrishnan et al., 2018). An imbalance of the microbiota, called dysbiosis, disturbs the anti-tumor immune response to immune checkpoint inhibitors (ICIs) (Gopalakrishnan et al., 2018; Rossi et al., 2019).

The reduction of gastric acidity secondary to PPIs leads to a decrease in the gastric bactericidal effect and a subsequent change in the gut microbiome. Bacteria that are naturally present in the oral cavity and usually destroyed in the gastric area emerge in the digestive tract (e.g., Streptococcaceae, Enterococcaceae) (Naito et al., 2018). The concentration of bacteria in the small intestine subsequently increases (e.g., *Salmonella*, *Campylobacter*, and C. difficile). Small intestinal bacterial overgrowth (SIBO) is the presence of 100,000 bacterial colonies/mL in the small intestinal content. PPI administration is considered a risk factor for SIBO (Kinoshita et al., 2018; Naito et al., 2018).

First, retrospective studies have not found statistically significant differences in the clinical activity of ICIs in terms of progression-free survival (PFS) and overall survival (OS) in different solid tumors (Mukherjee et al., 2018; Zhao et al., 2019). Two retrospective analyses of two randomized control trials found a major impact on survival (Chalabi et al., 2020; Hopkins et al., 2020). The first was Chalabi’s study (pooled POPLAR and OAK studies), which found reduced OS and PFS in patients with advanced or metastatic non-small cell lung cancer treated with atezolizumab and concomitant PPIs compared with survival in non-PPI recipients (HR 1.45, 95% CI 1.20–1.75, *p* = 0.0001 and HR 1.30, 95% CI 1.10–1.53, *p* = 0.001, respectively) (Chalabi et al., 2020). Similar results were found by Hopkins et al. in advanced or metastatic urothelial cancer treated with atezolizumab (HR 1.52, 95%CI 1.27–1.83, *p* < 0.001 and HR 1.38, 95% CI 1.18–1.62, *p* < 0.001) (Hopkins et al., 2020).

Two recent meta-analyses reported that PPI use was not associated with reduced survival in patients undergoing ICI treatment (Li et al., 2020a; Li et al., 2020b). However, these meta-analyses included only five and seven studies. Since then, many studies have continued to explore this DDI, and Table 5 summarizes the latest ones (Mukherjee et al., 2018; Zhao et al., 2019; Chalabi et al., 2020; Cortellini et al., 2020; Hopkins et al., 2020; Iglesias-Santamaría, 2020; Buti et al., 2021; Cortellini et al., 2021; Gaucher et al., 2021; Jun et al., 2021; Rounis et al., 2021; Ruiz-Bañobre et al., 2021). Robust recommendations for PPI use cannot be inferred given the retrospective nature of the currently available evidence, but caution should be exercised with ICIs (Rossi et al., 2019; Hussain et al., 2021). Further prospective studies on ICI and PPI DDIs are warranted.

**TABLE 5:** Main studies on interaction between immune checkpoint inhibitors and co-medication with proton pump inhibitors.

Location	Type of study	ICI	PPI intake definition	Number of patients	Results	References
NSCLC	Retrospective monoleft	Not specified	PPIs within 1 month before or after the first dose of ICI	Users = 40 (37%) *vs.* non-users = 69 (63%)	No difference on PFS (*p* = 0.343) or OS (*p* = 0.754)	Zhao et al. (2019)
NSCLC	Retrospective analysis (pooled data from POPLAR and OAK)	Atezolizumab	PPIs within 30 days before or after the first dose of ICI	Users = 234 (31%) in atezolizumab group vs. non-users = 523 (69%)	Reduced OS (HR 1.45, 95% CI1.20–1.75, *p* = 0.0001) and PFS (HR 1.30, 95%CI 1.10–1.53, *p* = 0.001)	Chalabi et al. (2020)
NSCLC	Prospective monoleft	Not specified	PPI intake ≥3 months before the initiation of ICI	Users = 23 (35%) vs. non-users = 43 (65%)	No difference on PFS (*p* = 0.062) and OS (*p* = 0.301)	Rounis et al. (2021)
NSCLC	Retrospective multileft	Pembrolizumab	Baseline exposure	Users = 474 (50%) *vs*. non-users = 476 (50%)	Reduced OS (HR = 1.49, 95% CI 1.26–1.77, *p* < 0.0001)	Cortellini et al. (2021)
HCC	Retrospective multileft	Any (91% single-agent anti-PD1)	PPIs within 30 days before the initiation of ICI	Users = 110 (35%) *vs.* non-users = 204 (65%)	No difference on OS (HR 0.98, 95% CI 0.71–1.36)	Jun et al. (2021)
Urothelial carcinoma	Retrospective analysis (individual-participant data from IMvigor210 and IMvigor211)	Atezolizumab	PPIs within 30 days before or after the first dose of ICI	Users = 471 (35%) *vs.* non-users = 889 (65%)	Reduced OS (HR 1.52, 95%CI 1.27–1.83, *p* < 0.001 reduced PFS (HR 1.38, 95%CI 1.18–1.62, *p* < 0.001)	Hopkins et al. (2020)
Urothelial carcinoma	Retrospective multileft	Single-agent anti-PD1 or anti-PDL1 (67% atezolizumab, 24% pembrolizumab)	PPIs within 30 days before the first dose of ICI	Users = 54 (45%) *vs.* non-users = 65 (55%)	Reduced OS (HR = 1.83, 95%CI 1.11–3.02, *p* = 0.02) and PFS (HR = 1.94, 95%CI 1.22–3.09, *p* = 0.005)	Ruiz-Bañobre et al. (2021)
Any (21% melanoma, 18% lung and others)	Retrospective monoleft	Single agent anti-PD1 or anti-PDL1 (46% nivolumab, 22% pembrolizumab, 32% other)	Not specified	Users = 73 (460%) *vs.* non-users = 85 (54%)	No difference on OS or PFS (*p* = 0.77)	(Mukherjee et al., 2018)
Any (52% NSCLC, 26% melanoma)	Retrospective multileft	Single-agent anti-PD1 or anti-PDL1 (61% nivolumab, 34% pembrolizumab)	Baseline exposure	Users = 491 (49%) vs. non-users = 521 (51%)	Reduced OS (HR 1.26, 95%CI 1.04–1.52, *p* = 0.0172) and PFS (HR 1.26, 95%CI 1.07–1.48, *p* = 0.005)	Cortellini et al. (2020)
Any (70% NSCLC)	Retrospective monoleft	Single-agent ICI (86% anti-PD1)	Baseline exposure	Users = 104 (48%) *vs.* non-users = 113 (52%)	Reduced OS (HR = 1.57, 95%CI 1.13–2.18, *p* = 0.0071)	Buti et al. (2021)
Any (55% NSCLC)	Retrospective multileft	Any (60% nivolumab, 25% pembrolizumab)	Not specified	Users = 78 (77%) *vs*. non-users = 23 (23%)	No reduced PFS (HR = 0.75, 95%CI 0.42–1.34, *p* = 0.346) or OS (HR = 0.79, 95%CI 0.40–1.56, *p* = 0.506)	Iglesias-Santamaría. (2020)
Any (45% NSCLC, 30% melanoma)	Retrospective monoleft	Any (61% nivolumab)	Baseline exposure or in the following 60 days	Users = 149 (40%) *vs*. non-users = 223 (60%)	No reduced OS (HR = 0.8, 95%CI 0.6–1.08, *p* = 0.148)	Gaucher et al. (2021)

HCC, hepatocellular carcinoma; HR, hazard ratio; ICI, immune checkpoint inhibitor; NSCLC, non-small cell lung cancer; OS, overall survival; PD1, programmed cell death 1; PDL1, programmed death-ligand 1; PFS, progression-free survival; PPI, proton pump inhibitors.

## Conclusion

**FIGURE 2:**
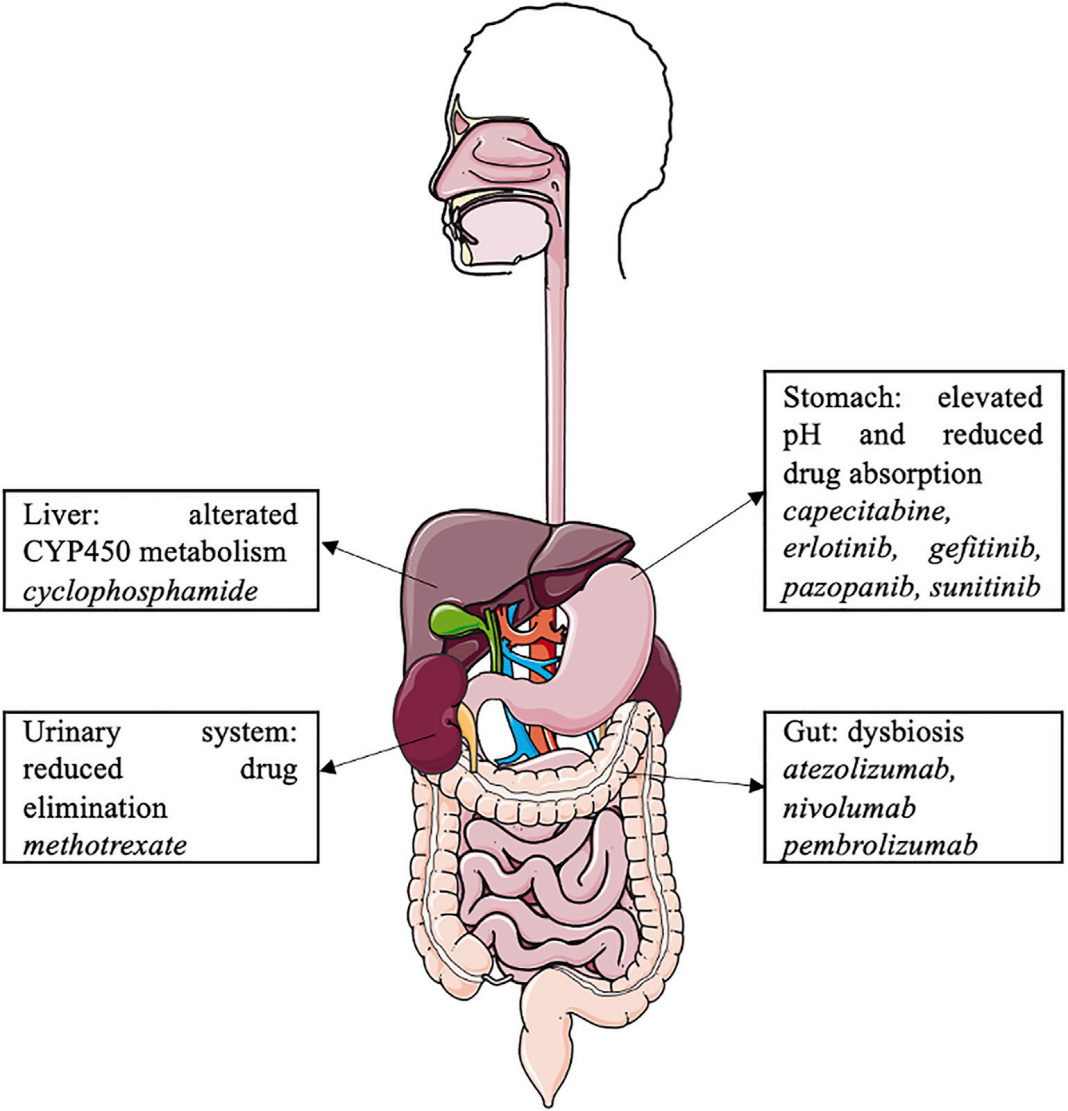
PPI-induced interactions between organ functions and anticancer drugs.

Proton pump inhibitors may interact with the cancer microbiome and various antineoplastic agents, such as oral and intravenous chemotherapy, tyrosine kinase inhibitors, and immune checkpoint inhibitors, and modulate their efficacy (Figure 2). However, due to the limitations of retrospective cohort studies with a small number of patients, data on these drug–drug interactions are limited, and further pharmaco-epidemiological studies are warranted. In the context of cancer-related treatment, oncologists should consider the pathophysiological consequences of PPI use, with significant drug–drug interactions and dysbiosis. PPIs should be dose appropriate and prescribed in accordance with the guidelines.
