# Supplementary material for: Proton Pump Inhibitors and Cancer: Current State of Play
Source: Front Pharmacol. 2022 Mar 14;13:798272. doi: 10.3389/fphar.2022.798272 (PMC8963837; doi:10.3389/fphar.2022.798272)
Supplement: Supplementary file 1 [file DataSheet1.docx]

Supplementary Material

# Supplementary Tables

## Table S1: Detailed list of terms

| **Proton pump inhibitors** | **Chemotherapy** | **Hormonotherapy** | **Targeted therapy** | **Tyrosine kinase inhibitors** |
| --- | --- | --- | --- | --- |
| esomeprazole | adriamycin | abiraterone | abemaciclib | afatinib |
| lansoprazole | bleomycin | anastrozole | bevacizumab | alectinib |
| omeprazole | capecitabine | enzalutamide | cetuximab | axitinib |
| pantoprazole | carboplatin | exemestane | everolimus | bosutinib |
| rabeprazole | cisplatin | fulvestrant | olaparib | brigatinib |
|  | cyclophosphamide | letrozole | palbociclib | cabozantinib |
|  | docetaxel | tamoxifen | panitumumab | ceritinib |
|  | doxorubicin |  | pertuzumab | cobimetinib |
|  | epirubicin |  | ribociclib | crizotinib |
|  | etoposide |  | talazoparib | dabrafenib |
|  | gemcitabine |  | trastuzumab | dasatinib |
|  | ifosfamide |  | ramucirumab | encorafenib |
|  | irinotecan |  |  | erlotinib |
|  | methotrexate |  |  | gefitinib |
|  | paclitaxel |  |  | ibrutinib |
|  | pemetrexed |  |  | imatinib |
|  | temozolomide |  |  | lapatinib |
|  | topotecan |  |  | lenvatinib |
|  | vinorelbine |  |  | lorlatinib |
|  |  |  |  | nilotinib |
|  |  |  |  | osimertinib |
|  |  |  |  | pazopanib |
|  |  |  |  | ponatinib |
|  |  |  |  | regorafenib |
|  |  |  |  | ruxolitinib |
|  |  |  |  | sorafenib |
|  |  |  |  | sunitinib |
|  |  |  |  | trametinib |
|  |  |  |  | vandetanib |
|  |  |  |  | vemurafenib |

## Table S2: List of terms for which the search was extended beyond five years

- axitinib

- bosutinib

- cobimetinib

- cyclophosphamide

- dasatinib

- head and neck squamous carcinoma

- erlotinib

- imatinib

- irinotecan

- methotrexate

- nilotinib

- osteoporosis

- pazopanib

- pharmacokinetic

- ponatinib

- sunitinib

- tyrosine-kinase inhibitors

- vandetanib
